# Transdifferentiation of pancreatic stromal tumor into leiomyosarcoma with metastases to liver and peritoneum: a case report

**DOI:** 10.1186/s12885-016-2976-8

**Published:** 2016-12-13

**Authors:** Chao Lin, Liping Wang, Jiyao Sheng, Dan Zhang, Lianyue Guan, Kai Zhao, Xuewen Zhang

**Affiliations:** 1 Department of Hepatobiliary and Pancreas Surgery, China-Japan Union Hospital of Jilin University, Changchun, 130033 China; 2 Department of Pathology, China-Japan Union Hospital of Jilin University, Changchun, 130033 China; 3 The Second Hospital of Jilin University, Ziqiang Street no. 218, Changchun, 130033 China

**Keywords:** Extra-gastrointestinal stromal tumor, Primary pancreatic leiomyosarcoma, Therapy, Diagnosis

## Abstract

**Background:**

Primary pancreatic leiomyosarcoma is a rare pancreatic malignancy; the clinical presentation and treatment is not well-characterized. Further, the molecular mechanisms underlying its pathogenesis are not known. We report a patient with pancreatic stromal tumor that progressed to primary pancreatic leiomyosarcoma with hepatic and peritoneal metastases.

**Case presentation:**

A 54-year-old woman was found to have pancreatic and hepatic tumor masses on routine health checkup. Owing to the difficulty in performing biopsy, this patient underwent open operation. Histopathological examination of pancreatic and liver biopsy specimen demonstrated spindle cells with nuclear mitoses. Immunohistochemical examination showed positive staining for Cluster of Differentiation117 (+) and negative staining for S-100 (-) and Smooth Muscle Actin (-). Thus, the patient was diagnosed as a case of advanced pancreatic stromal tumor with liver metastases. After surgery, treatment with oral imatinib mesylate combined with thymosin injection therapy was prescribed. Follow-up examination at 13-months revealed multiple nodular masses in liver and right peritoneum. The patient underwent a second surgery. Liver biopsy and the resected peritoneal specimen showed positive staining for Discovered On Gastrointestinal tumor-1(weak +), Actin (+), Smooth Muscle Actin (+) and negative staining for Cluster of Differentiation117 (-) Cluster of Differentiation34 (-) and S-100 (-). Histopathological examination showed spindle cells with nuclear mitoses. The final diagnosis was primary pancreatic leiomyosarcoma, transdifferentiating from pancreatic stromal tumor, with liver and peritoneal metastases.

**Conclusions:**

Surgery is the first line treatment for primary pancreatic leiomyosarcoma and extra-gastrointestinal stromal tumors. In the present case, radical resection was not performed owing to hepatic metastases. Palliative treatment with radioactive ^125^I ion implantation and microwave coagulation therapy was administered. However, the long-term therapeutic effect needs to be assessed in future.

**Electronic supplementary material:**

The online version of this article (doi:10.1186/s12885-016-2976-8) contains supplementary material, which is available to authorized users.

## Background

Primary pancreatic leiomyosarcoma is a rare pancreatic tumor, which accounts for approximately 0.1% of all primary pancreatic malignant neoplasms [1]. However, very little is known about the tumor [2]. In this case report, we present our experience with a case of pancreatic stromal tumor, which progressed to primary pancreatic leiomyosarcoma with liver metastases.

## Case presentation

**Fig. 1:**
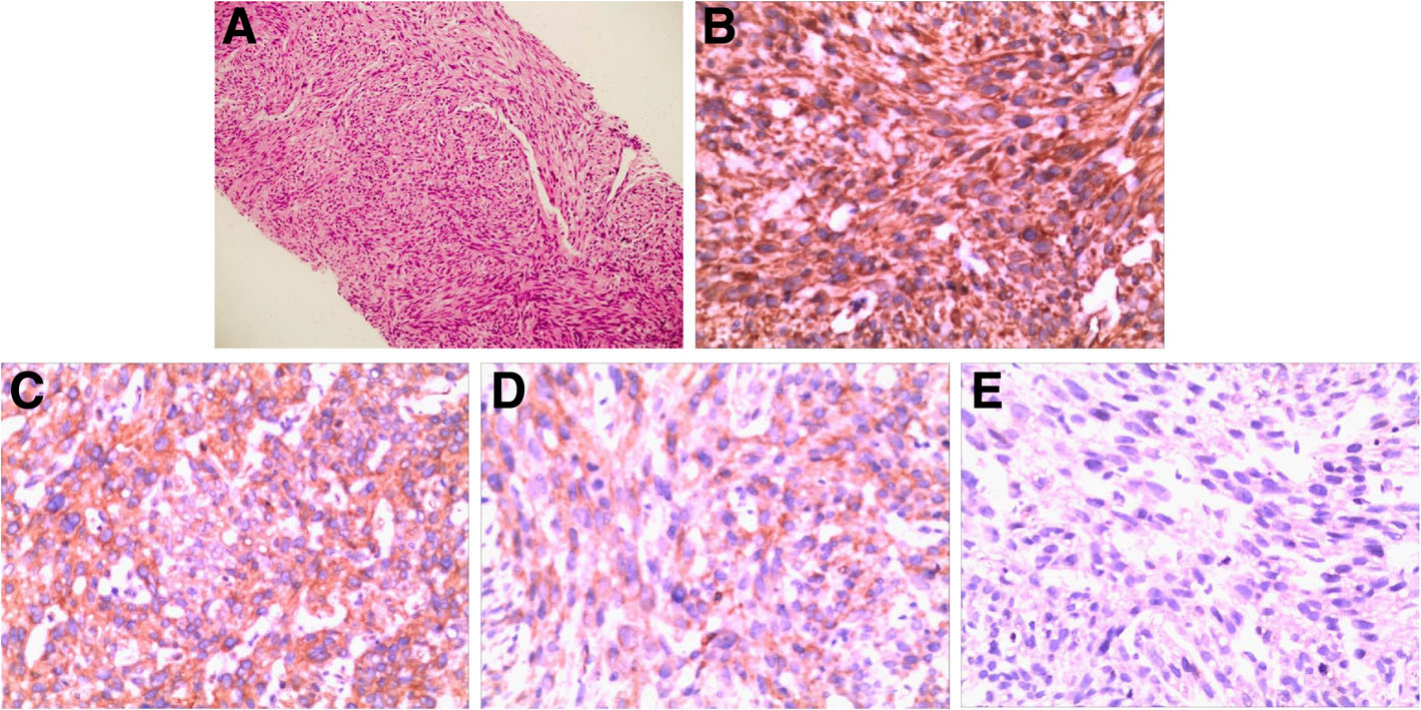
Histopathological (**a**) and immunohistochemical examination of the first biopsy specimen (**b**, **c**, **d**, **e**) showing Vimentin (+), DOG-1 (+), CD117 (+), SMA (-), respectively

A 54-year-old woman was found to have pancreatic and hepatic tumor masses on a routine health check-up. Abdominal ultrasound showed a clearly delineated hypoechoic mass (2.4 cm × 2.0 cm) in the IVa segment of the left hepatic lobe, near the diaphragmatic dome and left hepatic vein. A pancreatic mass (4.6 × 2.2 cm) located on the dorsal aspect of pancreatic body, in front of splenic vein, was also observed (Additional file 1: Figure S1A-B). Hepatic contrast ultrasound revealed a significant increase in size of the mass in the left liver lobe during the arterial phase. Abdominal computed tomography (CT) revealed a heterogeneous low-density mass with an ill-defined swelling in the pancreas (Additional file 1: Figure S1C-F). Owing to the difficulty in performing biopsy, open operation was performed. Intraoperative macroscopic findings included a gray hard pancreatic mass in the middle segment of the pancreas and a mass in the left liver lobe. Fine needle aspiration cytology showed atypical cells. The diagnosis of pancreatic cancer could not be excluded. Radioactive ^125^I ion implantation for the pancreatic tumor mass, and microwave coagulation therapy for the hepatic lesions was administered. Histopathological examination of pancreatic and liver biopsy specimens demonstrated spindle cells with nuclear mitoses (1-2 per 50× high power field) (Fig. 1a). Immunohistochemical examination showed positive staining for Vimentin (+) (Fig. 1b), Discovered On Gastrointestinal tumor (DOG)-1(+) (Fig. 1c), Cluster of Differentiation (CD) 117 (+) (Fig. 1d), 60%Ki67 (+), and negative staining for S-100 (-), CD34 (-), Cytokeratin (-), Smooth Muscle Actin (SMA) (-) (Fig. 1e), Desmin (-), and EMA (-). A diagnosis of advanced pancreatic stromal tumor with liver metastases was made.

The patient recovered well after surgery. Oral imatinib mesylate (300 mg once a day) plus thymosin subcutaneous injection (1.6 mg twice a week for 4 weeks) were prescribed. Follow-up CT and ultrasound examinations at 6 and 10 months showed no signs of tumor growth in the pancreas and liver (Additional file 2: Figure S2A-D). Repeat CT at the 13-month follow-up revealed multiple hepatic nodular masses in IVa segment, V segment and the border of V and VI segments (size 0.3–0.5 cm) and right peritoneum (Additional file 2: Figure S2E-F).

**Fig. 2:**
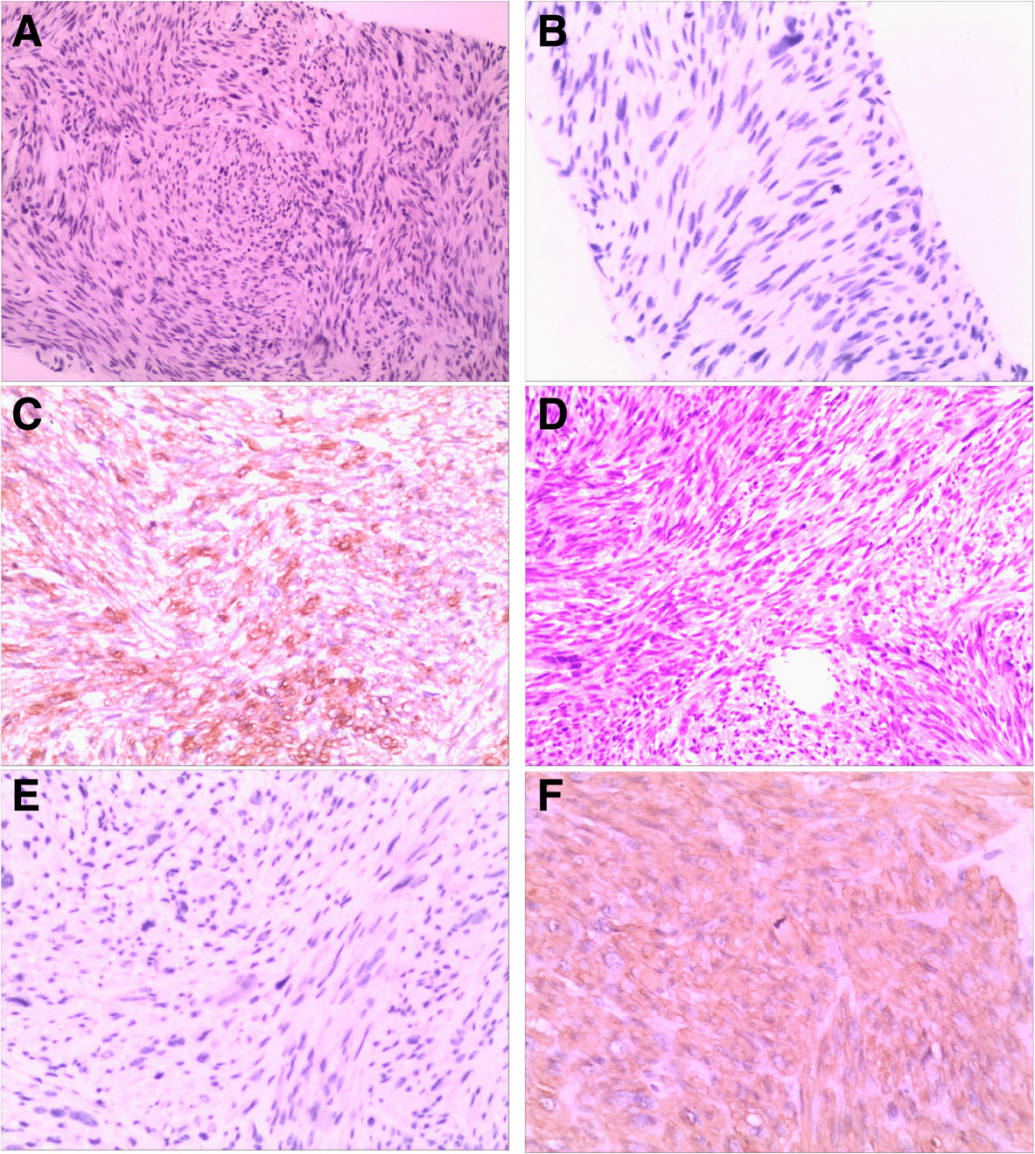
Histopathological and immunohistochemical examination of liver biopsy specimen (**a**-**c**) and surgically resected peritoneal deposits (**d**-**f**) showing spindle cells with nuclear mitoses, CD117 (-), SMA (+) respectively

A second surgery was performed to remove the peritoneal mass and to obtain liver biopsy. During operation, microwave coagulation therapy for liver lesions was re-administered. Immunohistochemical study of liver biopsy specimen and the resected peritoneal specimen showed positive staining for DOG-1 (weak +), Actin (+), SMA (+) (Fig. 2c and f), Caldesmon (+), Ki67 (30% +) and negative staining for CD117 (-) (Fig. 2b and e), Desmin (-), CD34 (-) and S-100 (-). Histopathological examination showed spindle cells with nuclear mitoses (14–20 per 10 high power fields) (Fig. 2a and d). Spindle shaped malignant cells with nuclear mitoses (2–5 per 10 high power fields) were also observed in the liver biopsy specimen.

The c-KIT and platelet-derived growth factor receptor α genes were sequenced. Wild-type variants were detected in exons 9, 11, 13 and 17 of the c-KIT gene and of exons 12 and 18 of the platelet-derived growth factor receptor α gene. The woman was finally diagnosed as a case of primary pancreatic leiomyosarcoma, which transdifferentiated from pancreatic stromal tumor, with liver and peritoneal metastases.

Leiomyosarcoma and its subtype, primary pancreatic leiomyosarcoma, have rarely been reported. The estimated worldwide incidence of PLMS is 1-2/100,000 population [3]. Baylor et al. found only five cases of primary pancreatic leiomyosarcoma in a study of 5057 patients with pancreatic cancer [4]. In 1951, Ross et al. reported the first case of primary pancreatic leiomyosarcoma [5]; since then, only 71 such cases are on record [3]. The gastrointestinal stromal tumor mostly arises from Cajal or its precursor cells [6, 7]. Histological and immunogenic properties of extra-gastrointestinal stromal tumors are similar to those of gastrointestinal stromal tumor, but the former originates from abdominal or retroperitoneal soft tissues [1]. To the best of our knowledge, this is the first report of a case of extra-gastrointestinal stromal tumor which progressed to primary pancreatic leiomyosarcoma.

The clinical manifestations are non-specific [8]. Our patient was asymptomatic prior to the diagnosis. The radiological examination for primary pancreatic leiomyosarcoma has low specificity [2]. Endoscopic ultrasonography-guided biopsy does facilitate a preoperative diagnosis; however, repeated sampling may be needed [6, 7].

Histological and immunohistochemical examination is the gold standard for diagnosis. In a study of 12 patients with primary pancreatic leiomyosarcoma, presence of more than 10/50 nuclear mitotic figures per high power field was associated with poor survival [9]. Xu et al. reported a median survival rate of 48 months; survival rates at 1, 3, 5 and 10 year were 66.6, 51.2, 43.9 and 29.3%, respectively [3]. Non-radical surgery and infiltration of surrounding organ and vessels were independently associated with poor prognosis. Another study demonstrated the presence of spindle shaped smooth muscle-like cells in primary pancreatic leiomyosarcoma, and IHC markers SMA (+), MSA (+), Desmin (+), CD117 (-), HMB45 (-), DOG-1 (-), CD34 (-) [10], while gastrointestinal stromal tumors were characterized by CD117 (+), CD34 (+), DOG-1 (+), SMA (-), Desmin (-), S-100 (-) [11].

Positive staining for CD117 may help differentiate between primary between extra-gastrointestinal stromal tumors and primary pancreatic leiomyosarcoma. In our study, initial immunohistochemical study of liver and pancreas specimens showed CD117 (+), while that of liver biopsy and peritoneal specimens at second- surgery detected CD117 (-) and SMA(+), which is indicative of transformation of extra-gastrointestinal stromal tumors to primary pancreatic leiomyosarcoma. Imatinib has been reported to induce differentiation of gastrointestinal stromal tumors into leimyoscarcoma [11], which may have contributed to the transdifferentiation observed in our patient.

## Conclusion

Surgery is the first line treatment for primary pancreatic leiomyosarcoma and extra-gastrointestinal stromal tumors. Radical resection either alone or in combination with protein receptor tyrosine kinase (RTK) inhibitor is associated with prolonged survival [12]. Radiofrequency ablation and liver transplantation are other potential therapeutic options in such patients; however, definitive evidence of their clinical efficacy is yet to be obtained. In the present study, the patient was diagnosed as pancreatic cancer with liver metastases after the first surgery. Thus, radical resection was not performed, and instead palliative treatment (radioactive ^125^I ion implantation and microwave coagulation therapy) was administered. In our patient, no obvious changes were observed in the pancreatic and hepatic tumors at 6 and 10-month follow-up. Taken together, we conclude that gastrointestinal stromal tumors with liver metastasis are amenable to local treatment plus imatinib therapy. The therapeutic efficacy of radioactive ^125^I ion implantation is yet to be documented. In addition, the long-term therapeutic outcomes need to be assessed in future.

## Additional files

Additional file 1: Figure S1. Preoperative abdominalultrasound (A, B) and computed tomography radiographs (C, D, E, F) showing tumor masses in liver and pancreas. (TIF 2890 kb)
Additional file 2: Figure S2. Follow-up ultrasound and abdominal CT radiographs at 6-month (A), 10-month (B, C, D) and 13-month follow-up (E, F) examination. *CT, computed tomography*. (TIF 2862 kb)

## Abbreviations

CT: Computed tomography
FNAC: Fine needle aspiration cytological
IHC: Immunohistochemical
PDGFRα: Platelet-derived growth factor receptor α
PLMS: Primary pancreatic leiomyosarcoma
RTK: Receptor tyrosine kinase
