# Supplementary figures and images for: Transdifferentiation of pancreatic stromal tumor into leiomyosarcoma with metastases to liver and peritoneum: a case report
Source: BMC Cancer. 2016 Dec 13;16:947. doi: 10.1186/s12885-016-2976-8 (PMC5154023; doi:10.1186/s12885-016-2976-8)

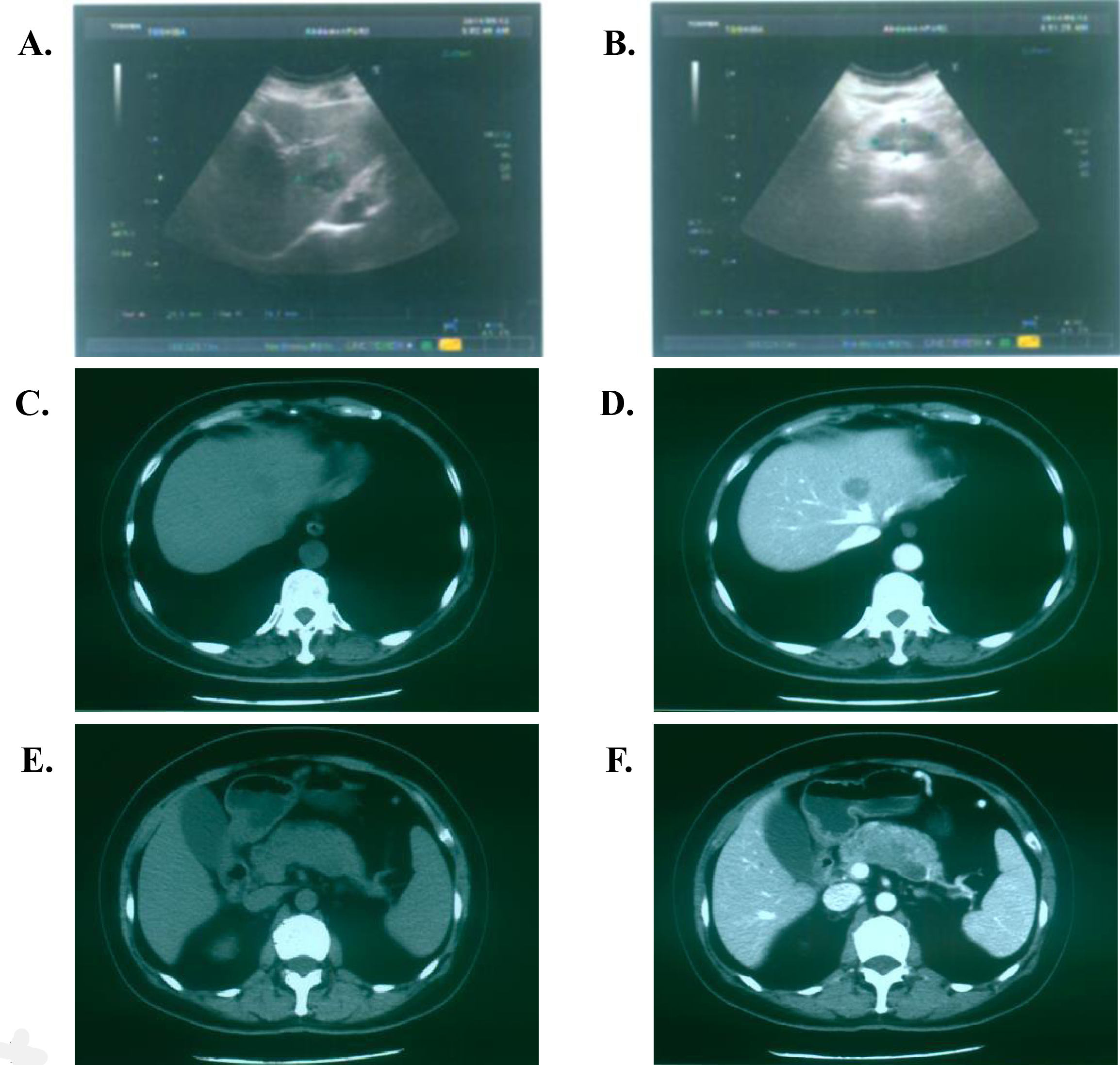

Supplement: Additional file 1: Figure S1. — Preoperative abdominalultrasound (A, B) and computed tomography radiographs (C, D, E, F) showing tumor masses in liver and pancreas. (TIF 2890 kb) [file 12885_2016_2976_MOESM1_ESM.tif]

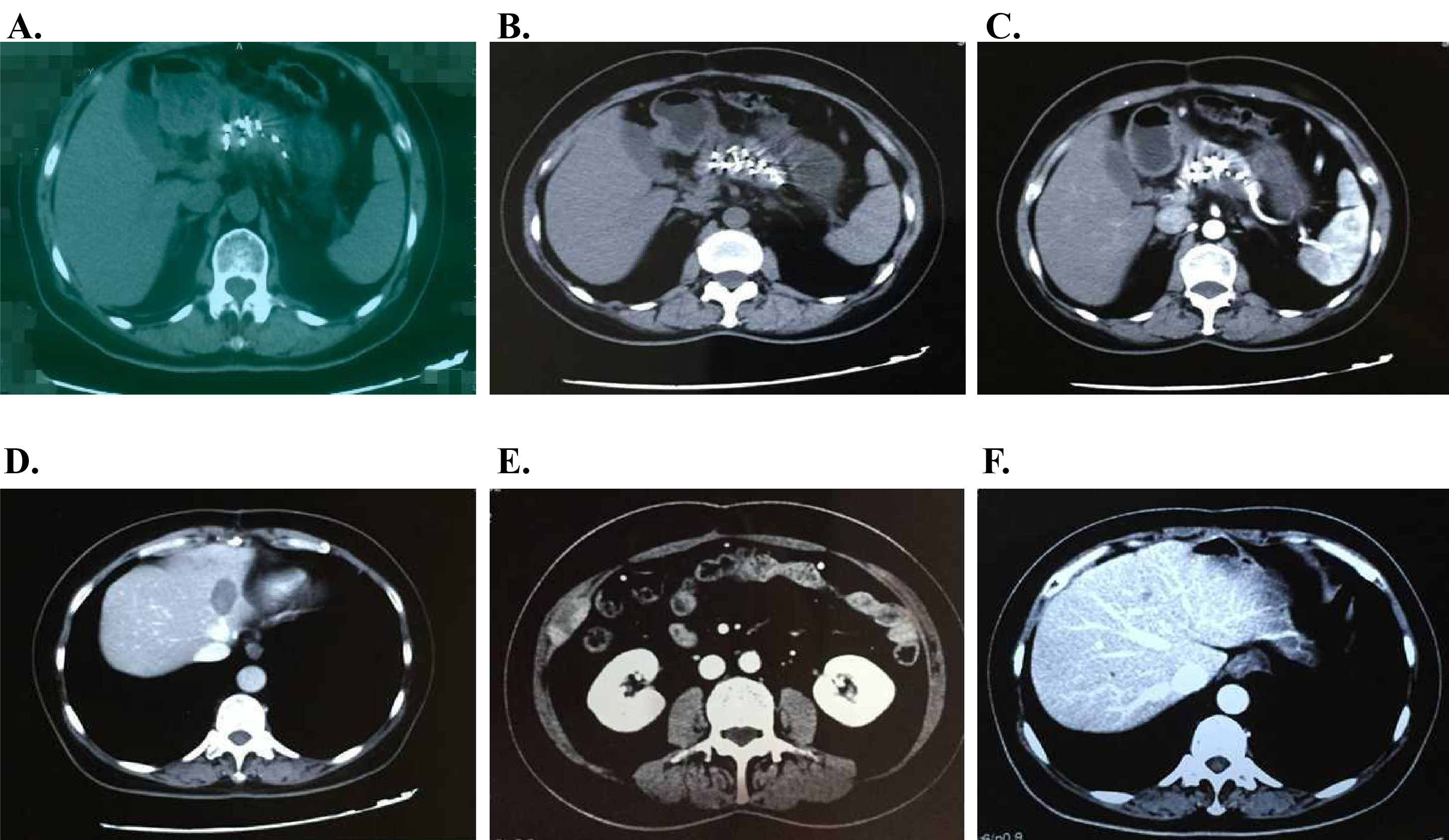

Supplement: Additional file 2: Figure S2. — Follow-up ultrasound and abdominal CT radiographs at 6-month (A), 10-month (B, C, D) and 13-month follow-up (E, F) examination. CT, computed tomography. (TIF 2862 kb) [file 12885_2016_2976_MOESM2_ESM.tif]
